# Five-to-five clear aligner therapy: predictable orthodontic movement for general dentist to achieve minimally invasive dentistry

**DOI:** 10.1186/s12903-021-02034-9

**Published:** 2021-12-29

**Authors:** Tommaso Weinstein, Giuseppe Marano, Raman Aulakh

**Affiliations:** 1 Humanitas Dental Center, Humanitas Research Hospital, Rozzano, MI Italy; 2 Private Practice, Rome, Italy; 3 Clear Aligner Diploma, City of London Dental School, Post Graduate Tutor Kings College, MSC Aesthetic Dentistry, London, UK

**Keywords:** Clear aligner therapy, Minimal invasive dentistry, Restorative dentistry, Digital impression, Interdisciplinary treatment planning

## Abstract

**Background:**

Esthetic dentistry has become a very important aspect of every dental treatment from the patient perspective, whether it is orthodontics or implant therapy. The aim of this article is to describe the advantages of a newly developed branch of five-to-five clear aligner therapy (CAT) (Invisalign Go, Align Technology, San Jose, Calif) in interdisciplinary treatments especially in terms of minimally invasive interventions.

**Case presentation:**

Two case reports are presented together with a comprehensive analysis using the SAFE (Safety, Assessment, Function, Ethics) assessment. This paper aims to introduce a new systematic in CAT. Invisalign Go (Align Technology, Santa Clara, California, USA) allows orthodontic treatment from second premolar and second premolar in both arches. It is specially designed for general practitioners devoted to restorative dentistry for a better planning of a multidisciplinary and mini-invasive treatment plan.

**Discussion and conclusion:**

The clinical results demonstrate how CAT is extremely useful in multidisciplinary treatment plan in order to straighten teeth especially in a pre-restorative phase to allow minimally invasive and adhesive restorations.

## Backgound

Esthetic dentistry has become a very important aspect of every dental treatment from the patient perspective, whether it is orthodontics or implant therapy [1]. In a recent survey study by Azarpazhooh et al. [2] questioning patient values related to treatment preferences for a tooth with apical periodontitis, esthetic outcome was the third among the most determining treatment preferences after communication/trust and retention.

Esthetics is not something separate from routine dentistry, indeed it “incorporates biological considerations to achieve the ideal function and emulate the pristine natural dentition, with a view to long-term performance and survival” [3].

Together with this, the relationship between patient and dentist has shifted from the paternalistic system to a new kind of relationship, a therapeutic alliance where the patient is able to make an informed decision based on scientifically based opinion made by his/her doctor [4].

In this perspective, new technologies allow dentist to show and inform the patient with a pre-visualization of the esthetic treatment. In restorative dentistry instruments such Digital Smile Design [5, 6] has changed the workflow throughout presenting treatment plan to patients.

Furthermore, minimally invasive dentistry, defined as “a systematic respect for the original tissue” [7, 8] has become a standard approach to solve dental pathologies.

This kind of approach is beneficial for esthetic treatment because it is possible to correct esthetical deficiencies with minimal interventions.

One of the most challenging situations is the correction of malpositioned teeth in adults from a restorative point of view.

To overcome this issue a valuable alternative is clear aligner therapy (CAT) which allows orthodontic movements by using removable thermoplastic appliances with modern CAD-CAM stereolithography and tooth movements simulation software [9]. It was introduced by Align Technology (Santa Clara, California, USA) in 1999 combining the concepts developed by Kesling [10], Ponitz [11] and McNamara [12].

CAT is better perceived by adult population than the conventional orthodontic therapies with braces because of esthetics.

The aim of this paper is to present two different cases where a specific CAT, treating only from second premolar to second premolar in both arches (Invisalign GO Align Technology, San Jose, Calif), was used in multidisciplinary treatment plans as a pre-restorative treatment in order to perform minimally invasive interventions.

## Cases presentation

All the cases were carefully evaluated with the SAFE assessment. SAFE assessment stands for *Stability Assessment Function and Ethics.* This assessment was created by one of the authors (Raman Aulakh) and used in other case reports [13, 14] in order to encompass orthodontics and restorative dentistry. It had to synchronize with Facially Generated Treatment Planning (FGTP) [15, 16]. The analysis for each case is reported in a dedicated table.

### Case 1

**Fig. 1:**
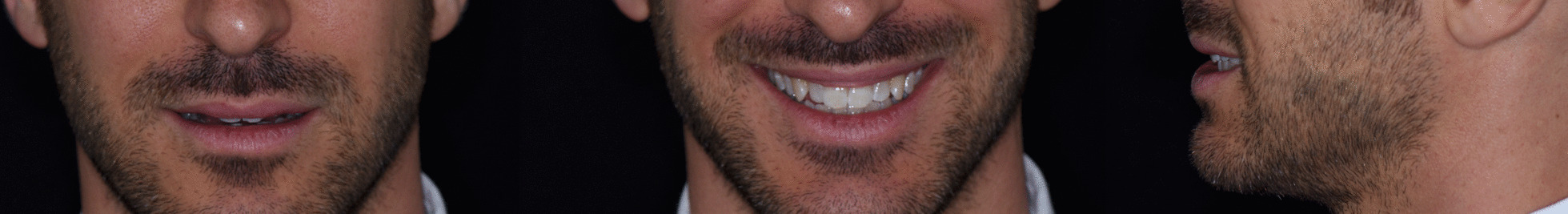
Case 1, initial situation. Extraoral views

**Fig. 2:**
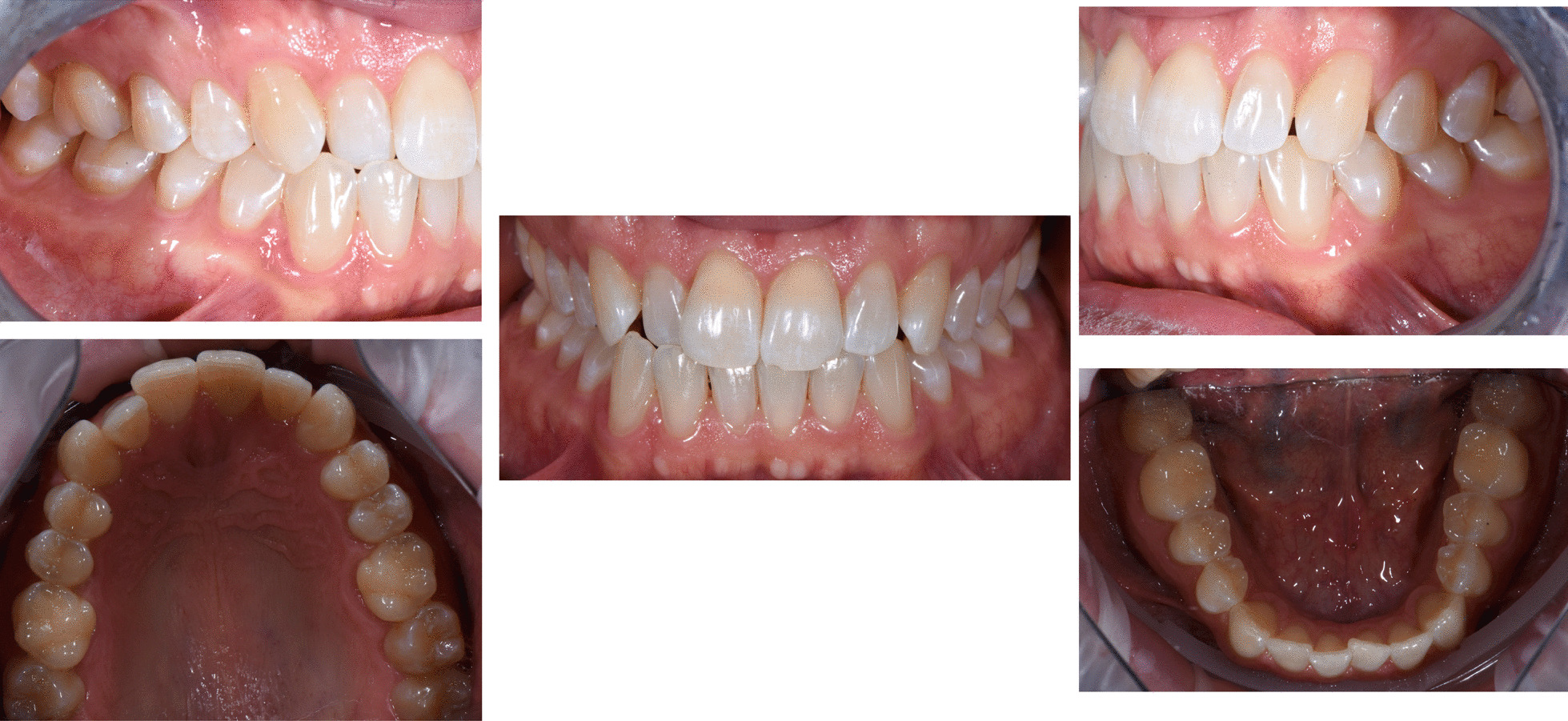
Case 1, initial situation, intraoral views

A 35 years old man complained about his broken upper left central incisor, upper right lateral cross-bite, diastema between 2.3 and 2.4 and crowding in the lower arch. There were no temporo-mandibular joint (TMJ) nor muscles signs of pathologies. The treatment plan aimed to correct, by means of clear aligner therapy, the misalignment, and fix the tooth 2.1 with a direct restoration. The clear aligner therapy was performed with Invisalign Go (Align Technology, San Jose, Calif) a system specifically designed for general practitioner involved in restorative dentistry with movements from second premolar to second premolar in both arches. The feasibility of the treatment is assessed through a dedicate app (Invisalign Photo Uploader) where a precise sequence of photos is uploaded (Figs. 1a–c and 2a–e).

The SAFE assessment of the case is reported in Table 1.

**Table 1:** Safe assessment case 1

Esthetics		Occlusion (static and functional)		Structure				Biology	
Face: frontal view		Sagittal		Status of teeth and restorations				Oral health	
Lower facial height	Normal	Overjet	2 mm	18		38		Oral Hygiene	Excellent
Upper midline to facial	Coincident	Incisor Classification	Class I	17		37		Phenotype	Thick
Lower midline to facial	Coincidetn	Molar relationship (rx—subdivision)	Class I—Full	16		36			
Chin point to facial	Coincident	Molar relationship (sx—subdivision)	Class I—Full	15		35			
**Face: Profile view**		Canine relationship (rx—subdivision)	Class I—Full	14		34			
Skeletal (severity)	Mild Class III	Canine relationship (sx—subdivision)	Class I—Full	13		33			
**Smile: analysis**		**Vertical**		12		32			
Smile line	Ideal	Overbite (%)	Decreased 20%	11		31			
Smile arc	Curved	Openbite	–	21	F	41			
Buccal corridors	Normal	**Transverse**		22		42			
**Soft tissue**		Crossbite	Yes—12	23		43			
Lip competence	Competent	Displacement	–	24		44			
Lip catch	Not present	Scissor Bite	–	25		45			
Naso-labial angle	Normal	**Space analysis**		26		46			
**Notes on esthetics:** The main concern of the patient is tooth 1.2 and diastema between tooth 2.3 and 2.4		Crowding upper	Mild (0-4 mm)	27		47			
		Crowding lower	Moderate (4–8 mm)	28		48			
		Spacing Upper	–	**Treatment goals:** Esthetics: Function: correction of crossbite tooth 1.2, correction of crowding lower arch Dentition: Restoring 2.1 Biology: no changes needed					
		Spacing Lower	–						
		**Arch form**							
		Upper	U shape						
		Lower	U shape						
		**Occlusal scheme**							
		Lateral excursion right	Canine						
		Lateral excursion left	Canine						
		Protrusive excursion	Incisor						
		**Tmd examination**							
		Pain on palpation	No						
		Clicks	No						
		Crepitus	No						
		Mouth opening	Regular						

Structure, legend: O = Restoration, X = Missing, A = Abraded, C = CARIES, F = fractured, R = Root filled, P = Perio involved

**Fig. 3:**
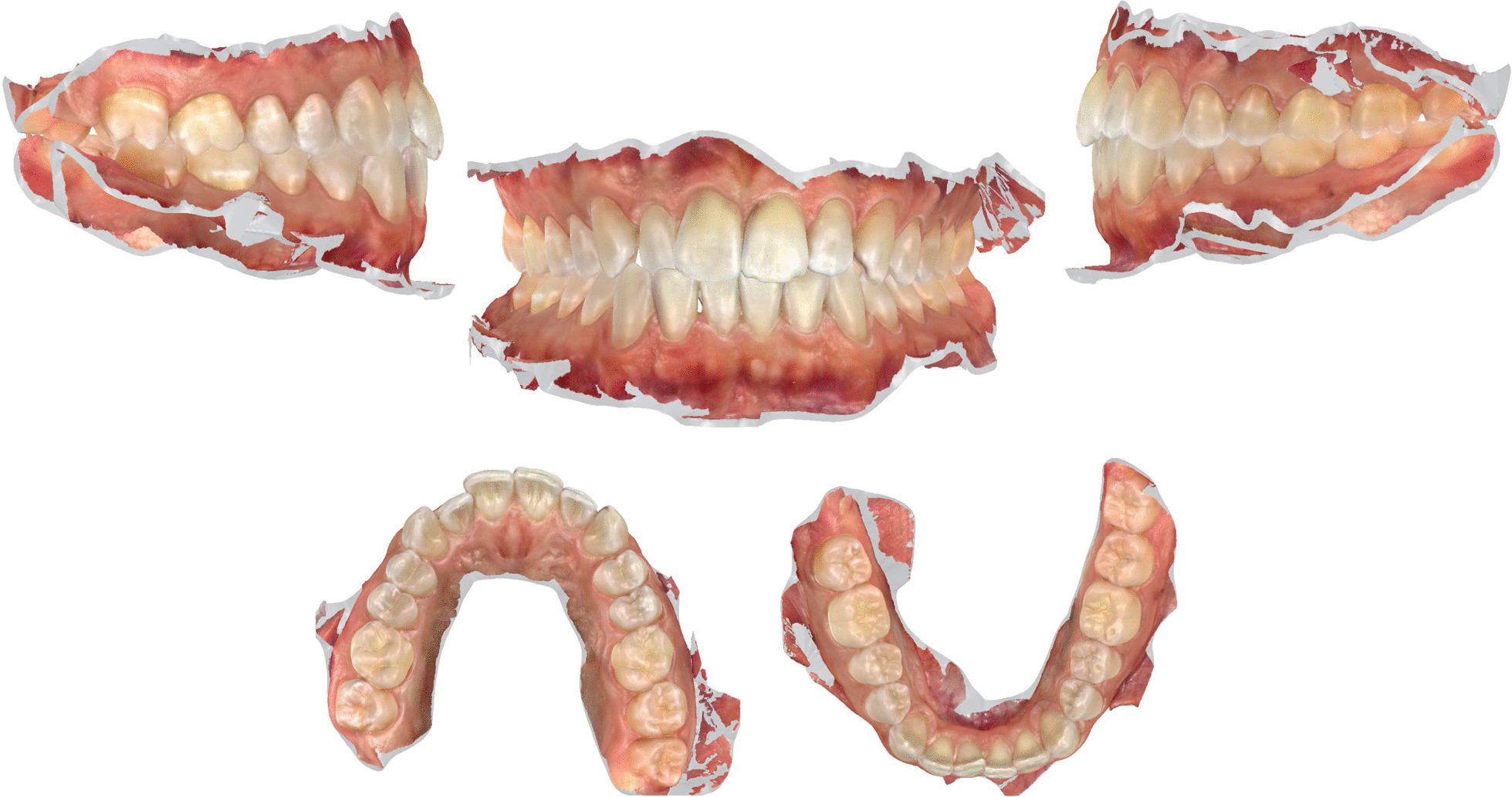
Case 1, initial situation, digital impression

**Fig. 4:**
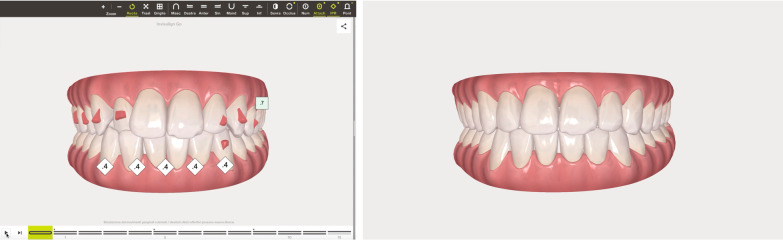
case 1, initial and final position planned on the Clin Check (Align Technology, San Jose, Calif)

**Fig. 5:**
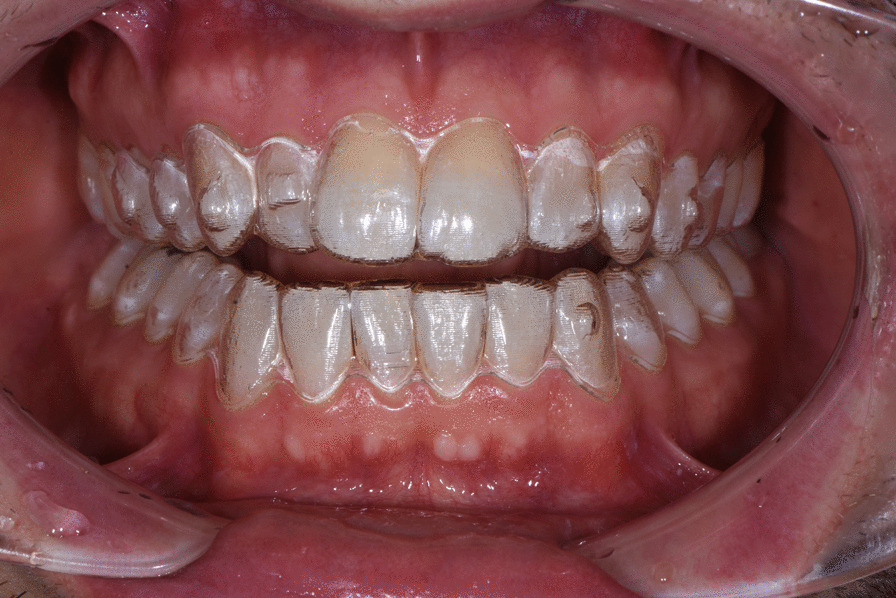
Case 1, delivery of the first set of clear aligners

**Fig. 6:**
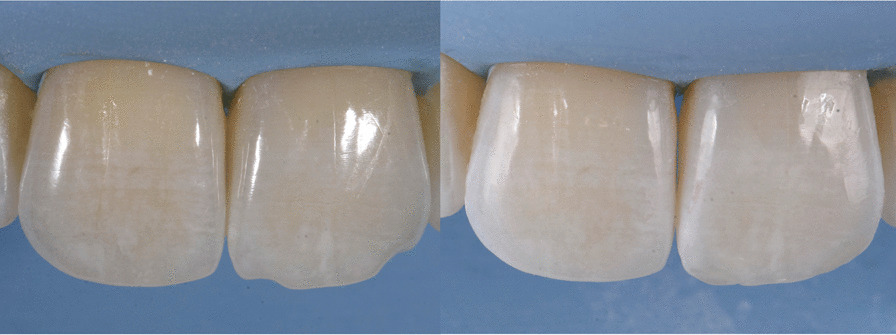
Case 1, direct restoration on tooth 2.1

**Fig. 7:**
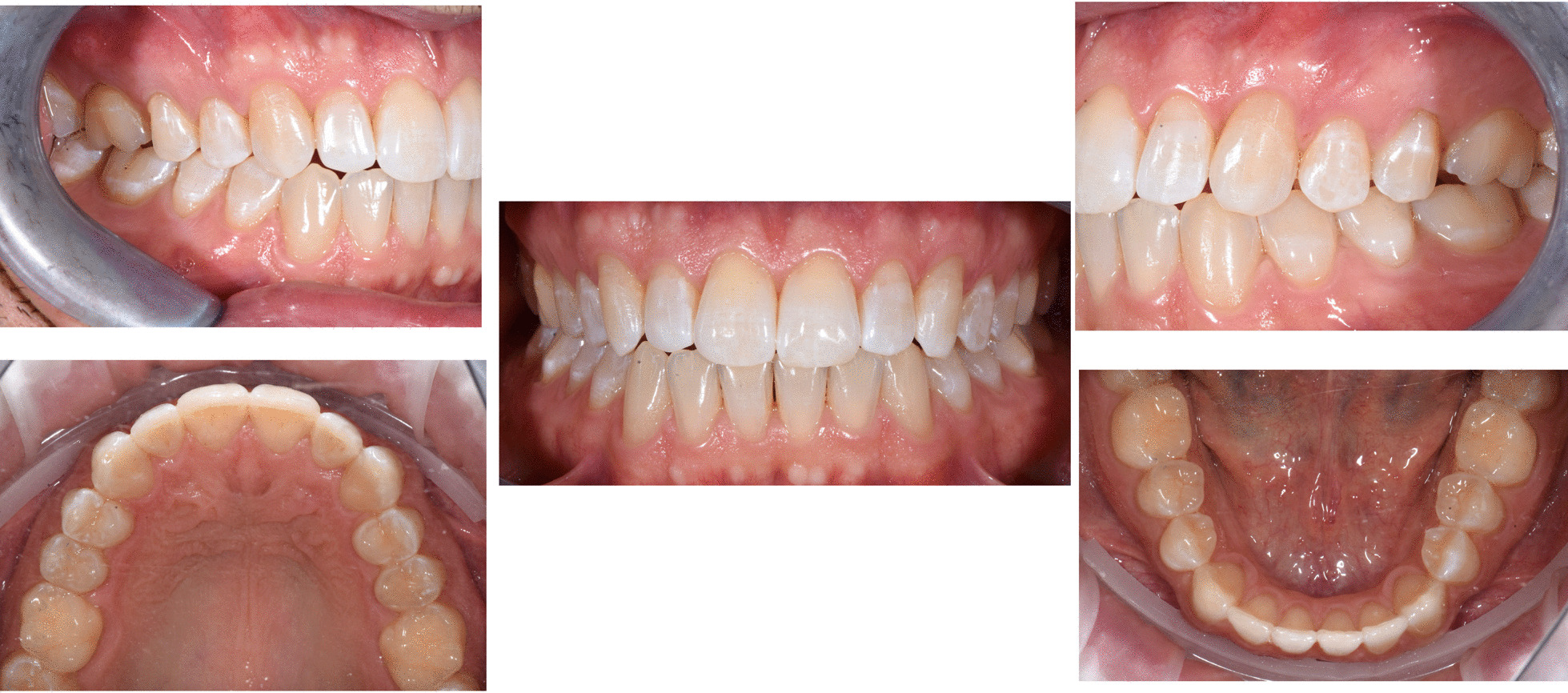
Case 1, final result, intraoral views

**Fig. 8:**
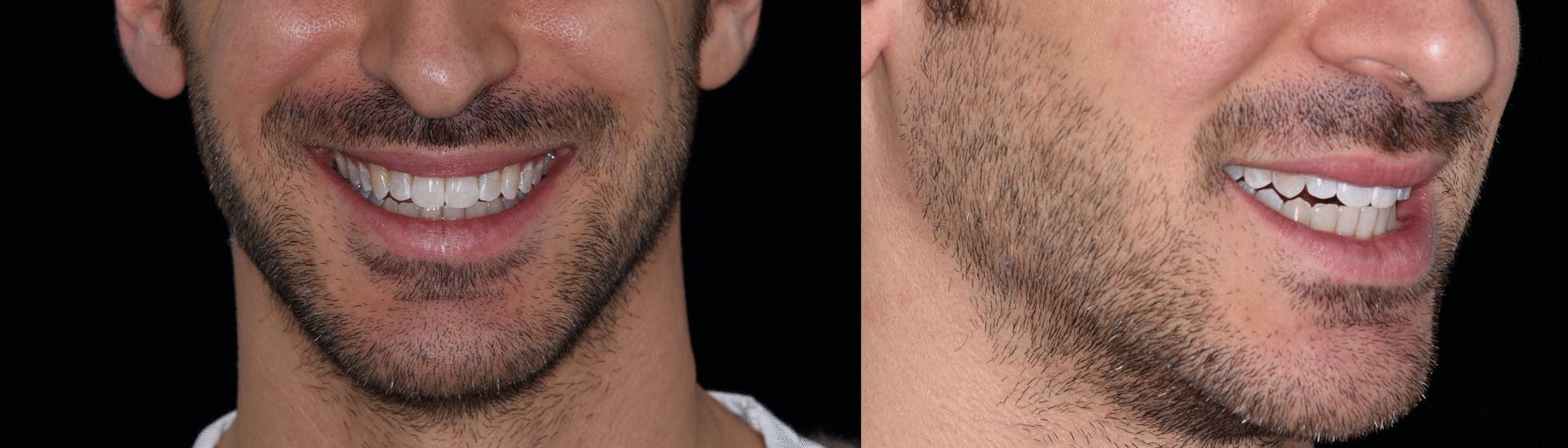
Case 1, final result, extraoral views

**Fig. 9:**
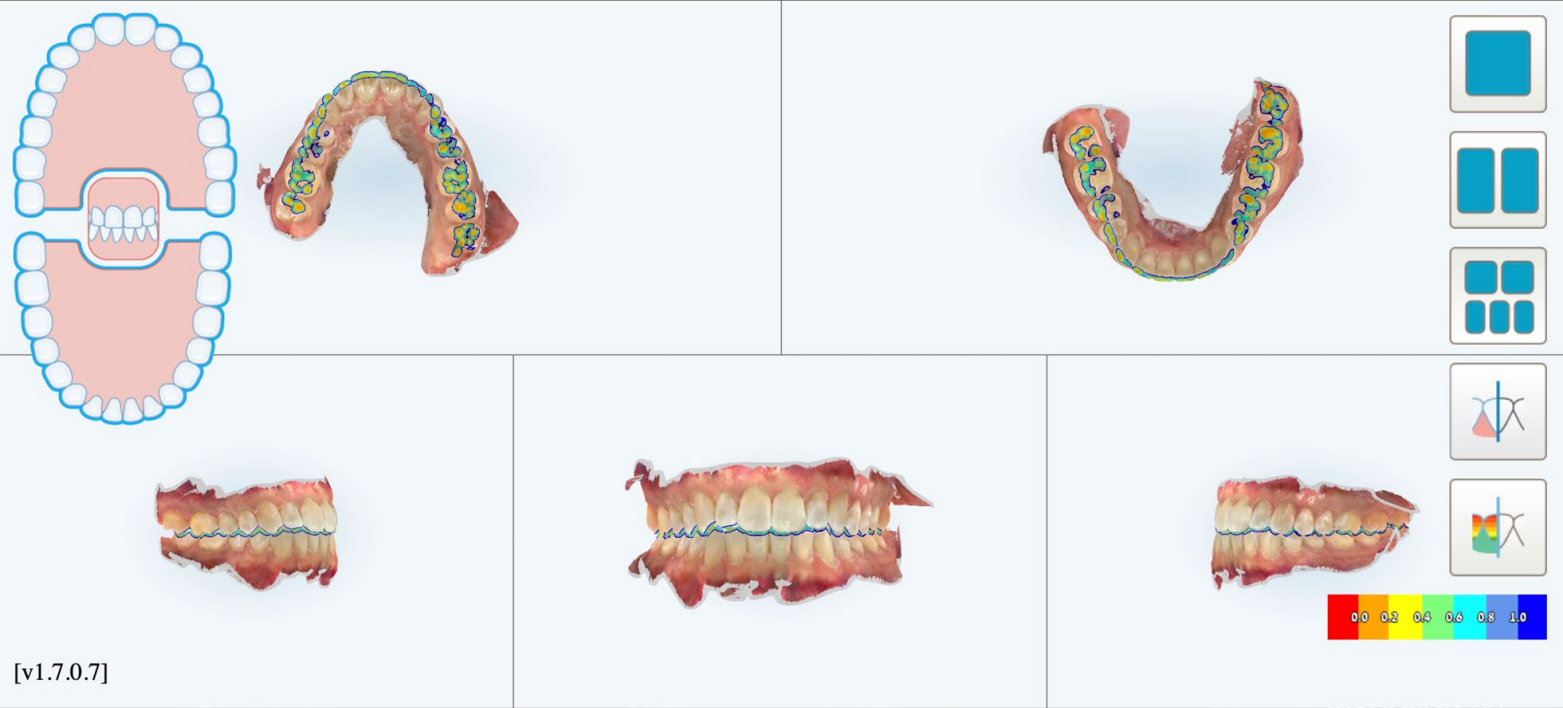
Case 1, digital impression of the final result

A digital impression (Fig. 3) was taken by means of an iTero scanner (Align Technology, San Jose, Calif) and sent for the Clin Check (Align Technology, San Jose, Calif; Fig. 4a, b), the software used for 3D alignment simulation. After the clinician had approved the Clin Check, the aligners were produced. Attachments were positioned and interproximal reduction was performed where needed following instructions of the manufacturer and a first set of aligners was positioned (Fig. 5). The therapy consisted of 12 aligners, patient was instructed to wear them at least 20 h a day, changing weekly, clinical control every 4 weeks. At the end of the treatment a new digital impression for a light refinement of the lower crowding was requested, for a total of 9 additive aligner with the same protocol. Cross bite, diastemas and crowding were resolved and direct restoration on tooth 21 was performed (Fig. 6a, b). Patient was happy with the final result (Figs. 7a–e and 8a, b) and a final digital impression (Fig. 9) was taken in order to have removable retainer (Vivera, Align Technology, San Jose, Calif) to be worn by night.

### Case 2

**Fig. 10:**
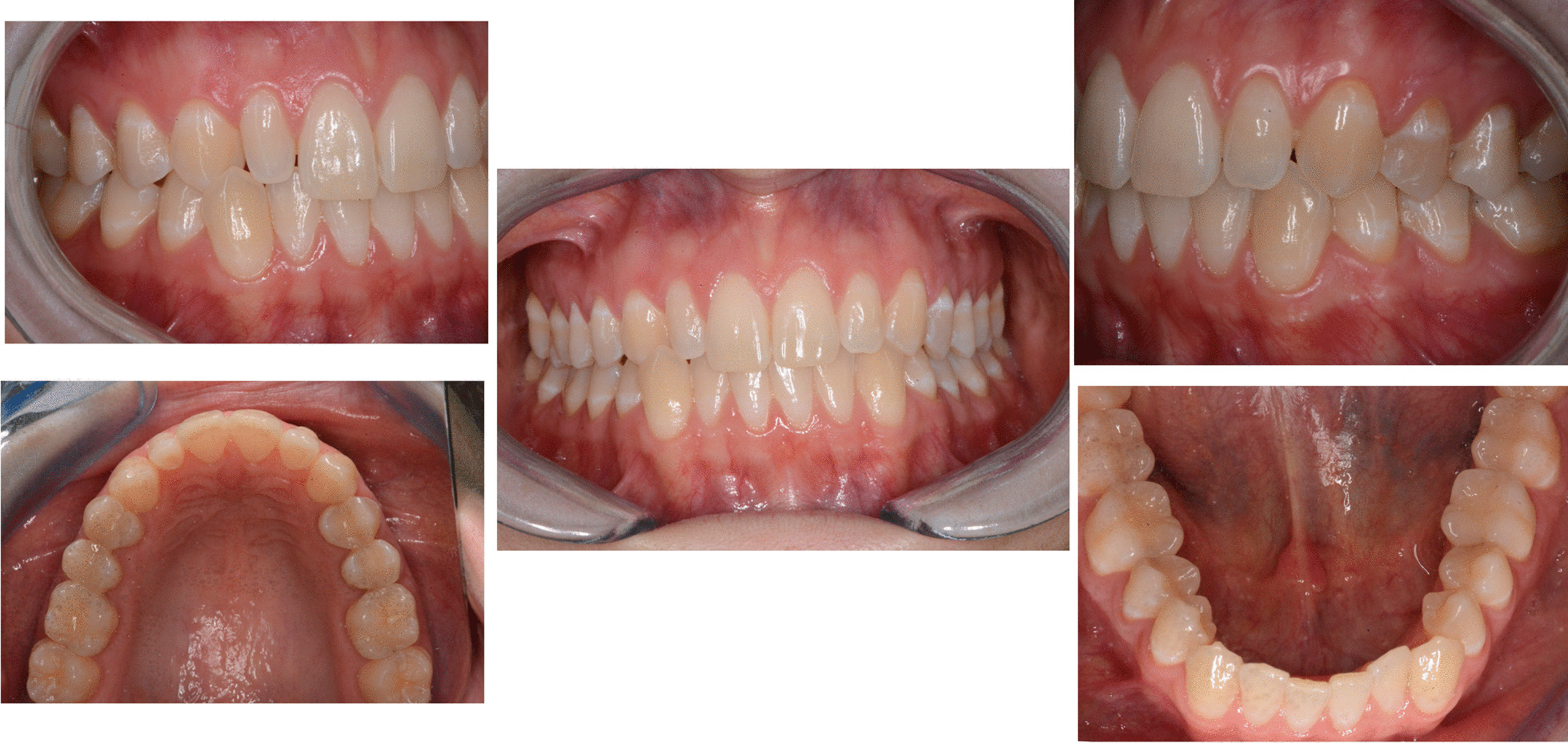
Case 2 initial situation. Intraoral views

**Fig. 11:**
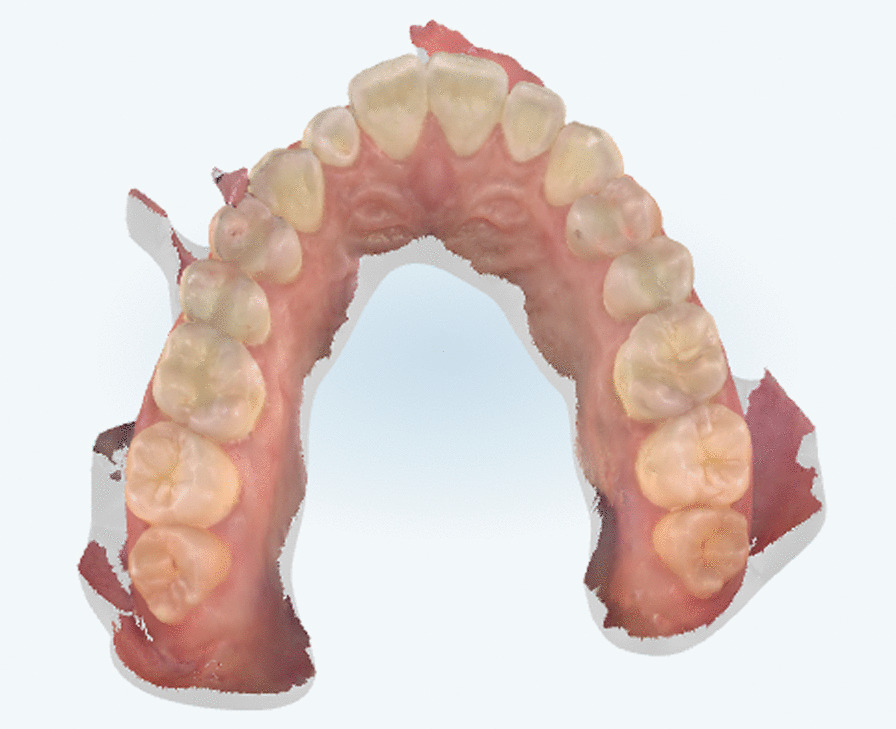
Case 2, digital impression upper arch

**Fig. 12:**
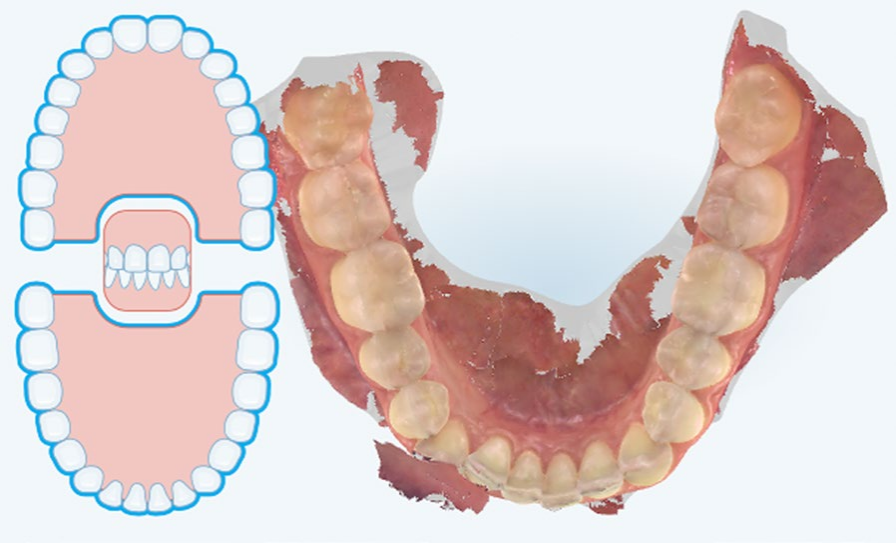
Case 2, digital impression lower arch

A 29 years old woman asked for consultation because she was near to her marriage, but unsatisfied about her smile. Clinical evaluation found a cross-bite of lower right canine and tooth discrepancy on upper right lateral incisor which was smaller than the contralateral (Figs. 10a–e, 11, 12). No signs of temporo-mandibular joint (TMJ) or muscles disease were detected. The SAFE assessment of the case is reported in Table 2.

**Table 2:** Safe assessment case 2

Esthetics		Occlusion (Static and Functional)		Structure				Biology	
**Face: frontal view**		**Sagittal**		**Status of teeth and restorations**				**Oral health**	
Lower facial height	Normal	Overjet	0 mm	18		38		Oral Hygiene	Excellent
Upper midline to facial	Coincident	Incisor Classification	Class I	17		37		Phenotype	Thick
Lower midline to facial	Coincident	Molar relationship (rx—subdivision)	Class I—Full	16		36			
Chin point to facial	Deviated	Molar relationship (sx- subdivision)	Class I—Full	15		35			
**Face: Profile view**		Canine relationship (rx—subdivision)	Class I—Full	14		34			
Skeletal (severity)	Class I	Canine relationship (sx—subdivision)	Class I—Full	13		33			
**Smile: analysis**		**Vertical**		12		32			
Smile line	Ideal	Overbite (%)	15%	11		31			
Smile arc	Curved	Openbite	—	21		41			
Buccal corridors	Normal	**Transverse**		22		42			
**Soft tissue**		Crossbite	1.3/4.3	23		43			
Lip competence	Competent	Displacement	—	24		44			
Lip catch	Not present	Scissor Bite	—	25		45			
Naso-labial angle	Normal	**Space Analysis**		26		46			
**Notes on esthetics:** The main concerns of the patient are teeth 1.3 and 4.3 in cross-bite, and tooth 1.2 that is conoid		Crowding upper	Mild (0-4 mm)	27		47			
		Crowding lower	Mild (0-4 mm)	28		48			
		Spacing Upper	—	**Treatment Goals:** Esthetics: create space for the restoration of 1.2 Function: correction of crossbite teeth 1.3/4.3, correction of lower arch crowding Dentition: Restoring 1.2 Biology: no changes needed					
		Spacing Lower	—						
		**Arch Form**							
		Upper	U shape						
		Lower	U shape						
		**Occlusal Scheme**							
		Lateral excursion right	Canine						
		Lateral excursion left	Canine						
		Protrusive excursion	Incisor						
		**Tmd Examination**							
		Pain on palpation	No						
		Clicks	No						
		Crepitus	No						
		Mouth opening	Regular						

Structure, legend: O = Restoration, X = Missing, A = Abraded, C = Caries, F = Fractured, R = Root filled, P = Perio involved

The treatment plan involved CAT to correct the cross bite and create a correct mesio-distal distance from 1.1 to 1.3 for a re-shaping of tooth 1.2 with a direct restoration.

**Fig. 13:**
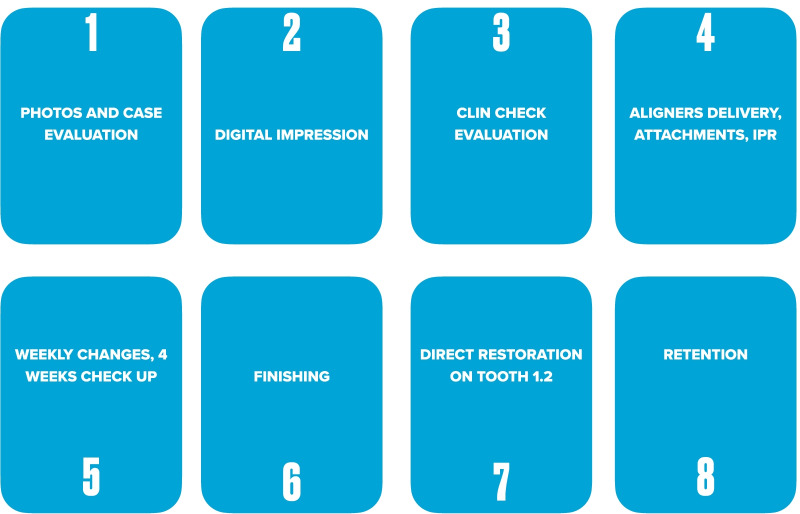
Workflow case 2

Patients disliked any solution with indirect restoration such as a veneer on tooth 1.2 which was proposed as an alternative treatment plan. The clinical procedure followed the pathways of Invisalign Go (Align Technology, San Jose, Calif) described in the previous case and detailed in Fig. 13.

**Fig. 14:**
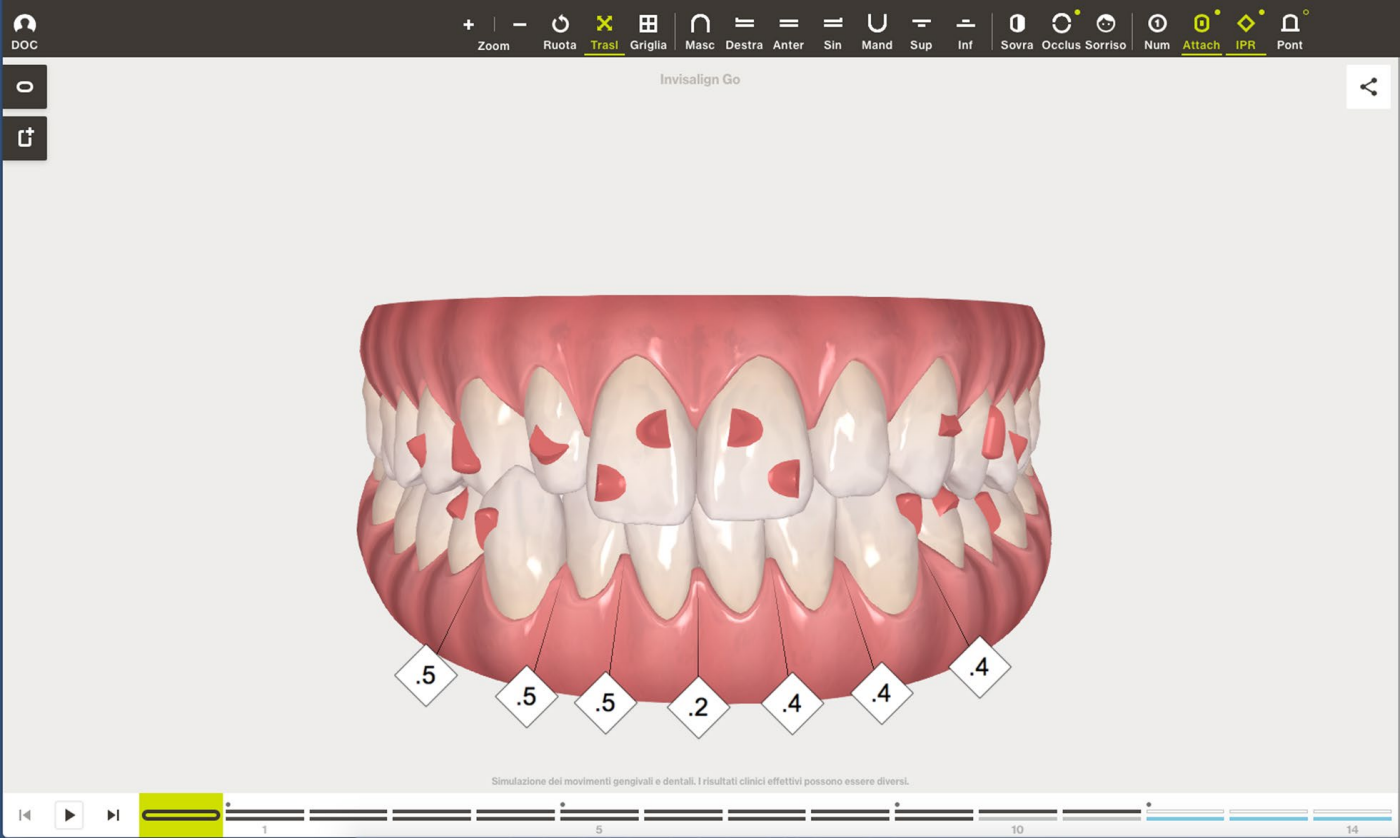
Case 2, initial position on the Clin Check (Align Technology, San Jose, Calif). The space for the restoration of 1.2 is carefully planned

**Fig. 15:**
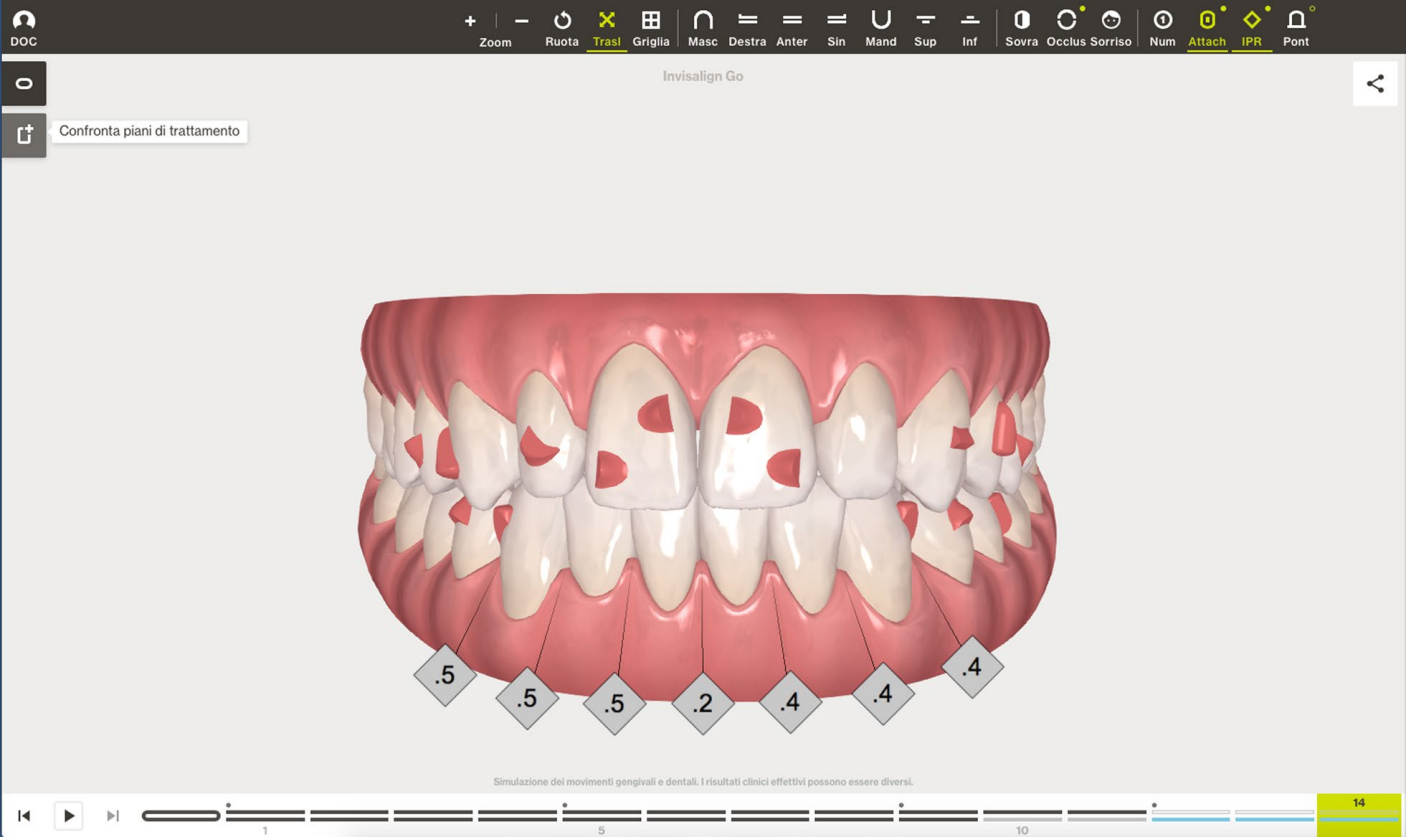
Case 2, final position on the Clin Check (Align Technology, San Jose, Calif)

**Fig. 16:**
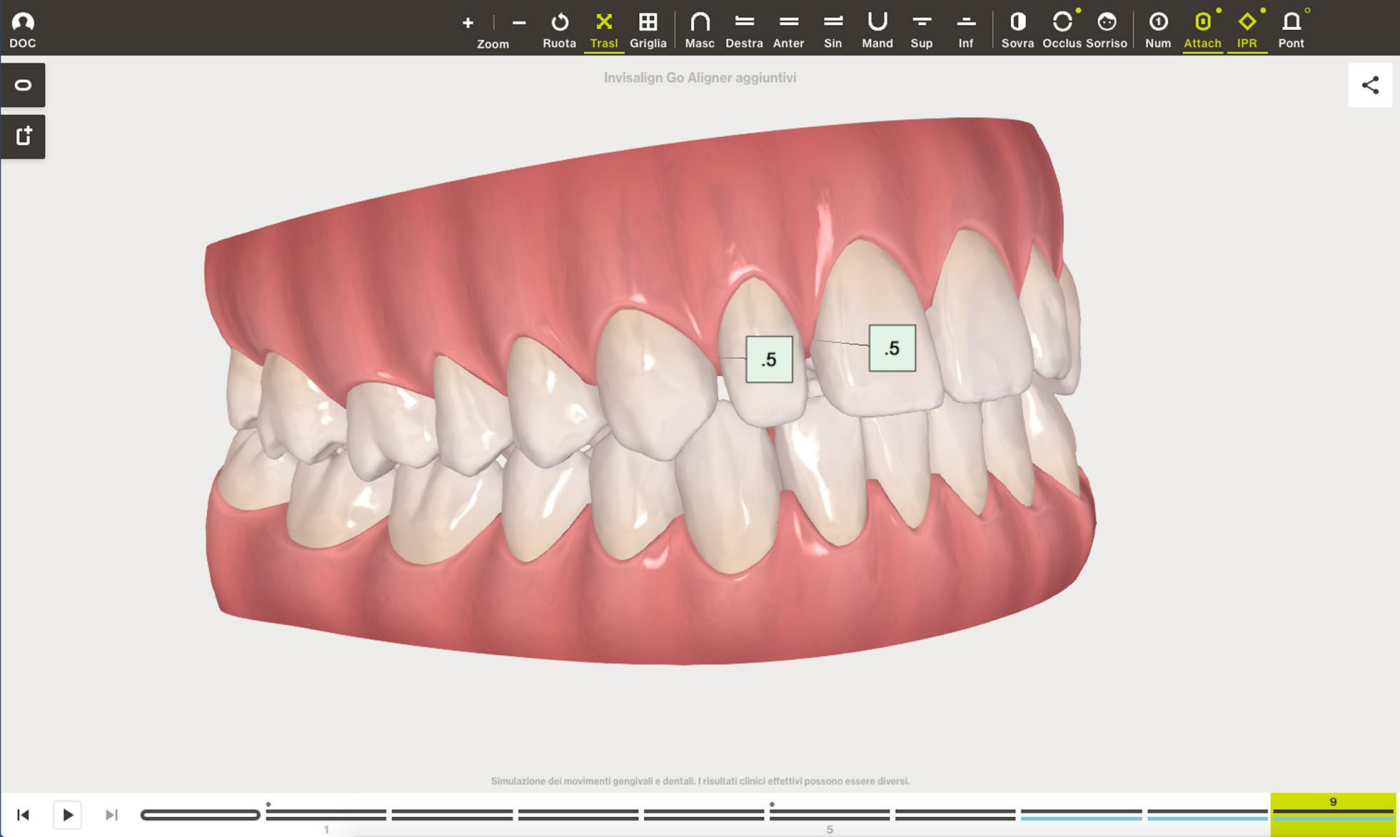
Case 2, the space for the restoration of 1.2 is carefully planned on the Clin Check (Align Technology, San Jose, Calif) during refinement

The Clin Check was carefully planned in order to correct the cross bite but also to create the correct space for tooth 1.2 which was calculated through Bolton analysis [17] with 14 phases. (Figs. 14, 15). Refinement was needed in order to improve the alignment and opening the adequate space for tooth 1.2 and consisted in 9 additional aligners (Fig. 16).

**Fig. 17:**
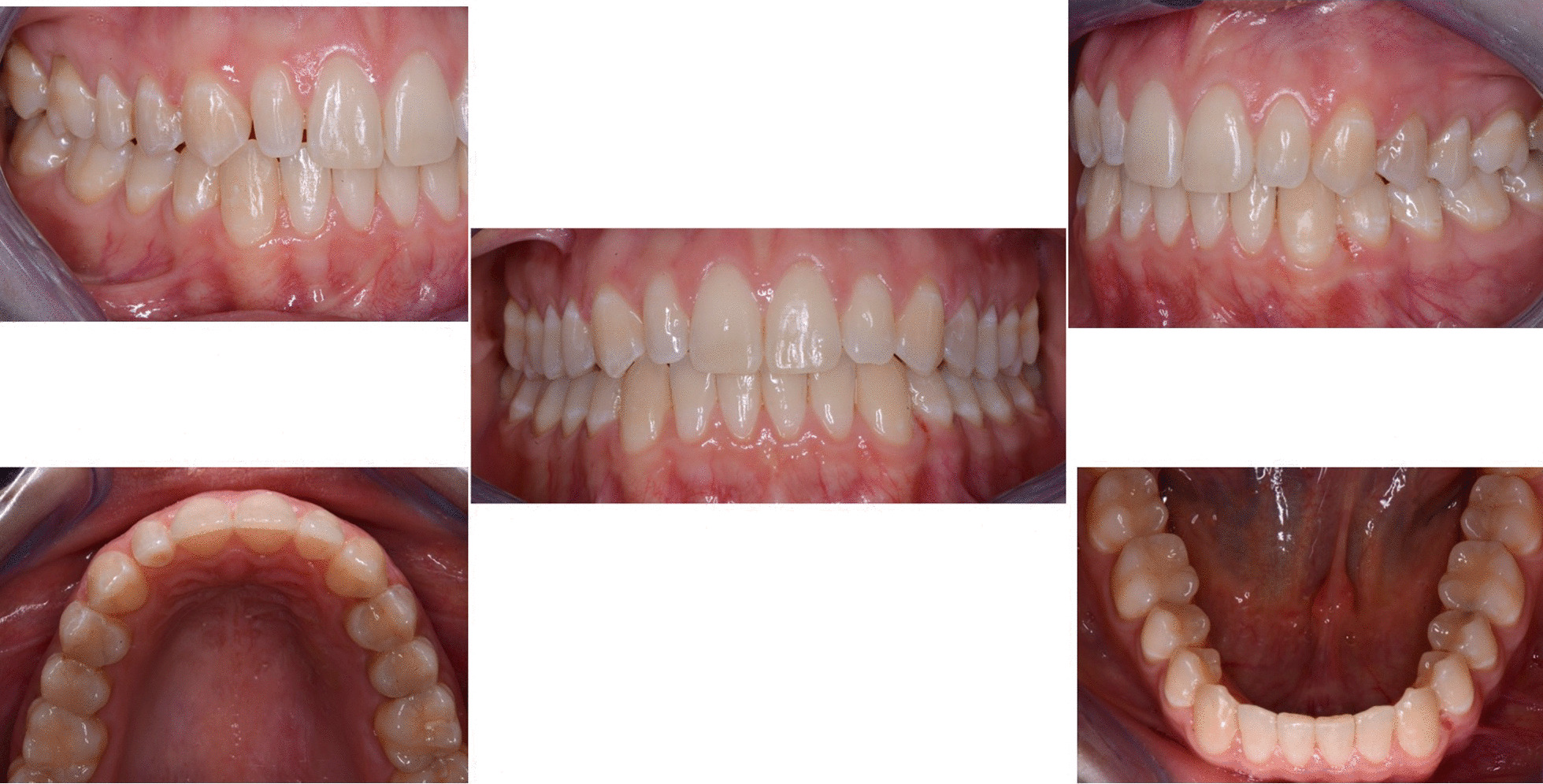
Final result case 2 after clear aligner therapy

**Fig. 18:**
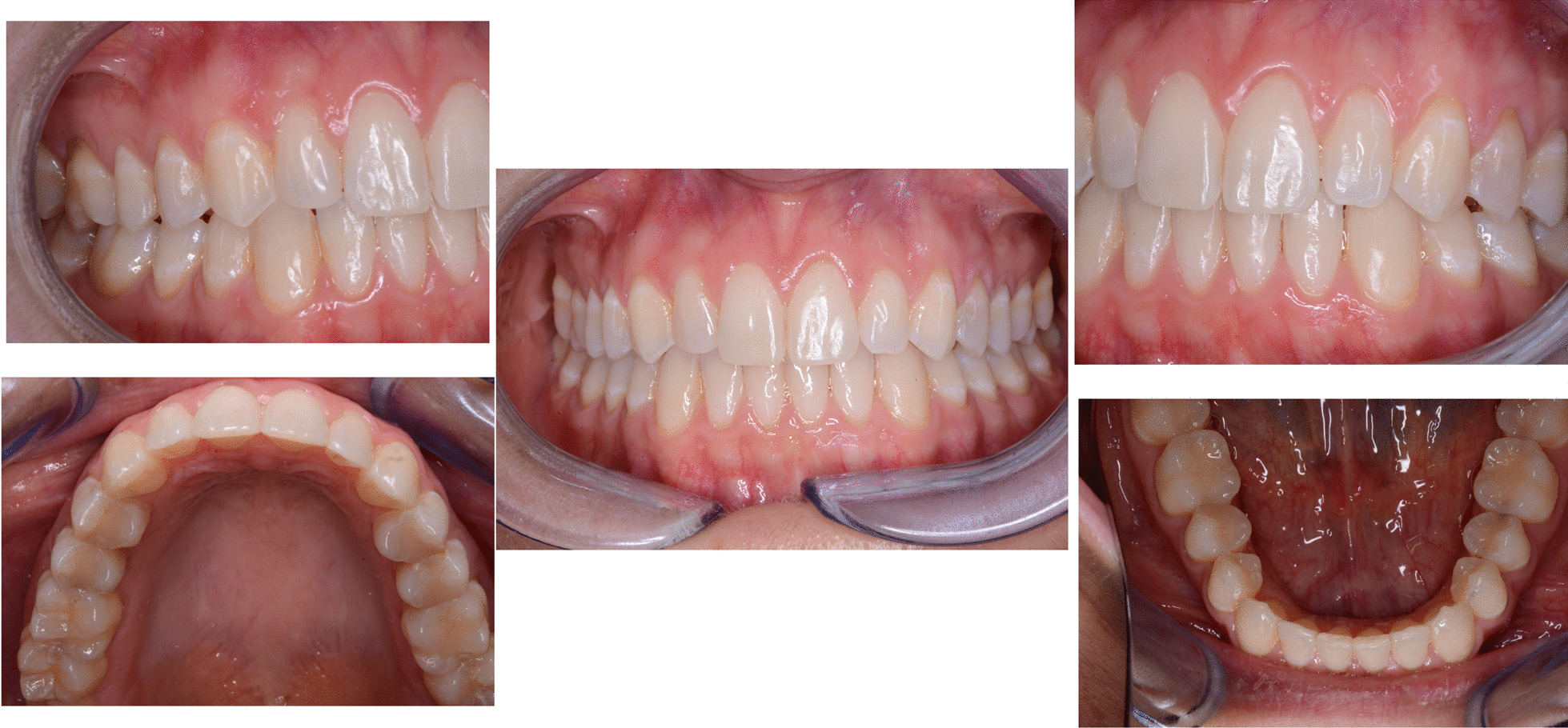
Final result case 2 after direct restoration on tooth 1.2. Intraoral views

Once the CAT was ended (Fig. 17a–e), a direct restoration on tooth 1.2 was made. Life-long retention protocol with Vivera Retainer (Align Technology, San Jose, Calif) was recommended (Fig. 18a–e).

## Discussion and conclusion

The patient evaluation in any complex rehabilitation should follow a pattern, a systematic approach which helps clinicians to elaborate the treatment plan [18–21]. Spear and Kokich elaborated the so called Facially Generated Treatment Planning (FGTP): the key reference point is an upper central incisor, and his position should be planned first in relation to face and lips [15, 16].

The FGTP uses the face to determine where the teeth, gingiva, and papillae should be positioned and then creating the rest of the treatment plan around the facially driven tooth position. The same that happens with a complete denture.

After determining the position of the central incisor and related esthetics there are three more steps to follow: function, structure and biology.

This sequence should always be followed to correctly plan the treatment; the operative sequence could otherwise be different. Esthetics has to be planned first in order to have a better final outcome and digital technologies could be very helpful [6].

**Fig. 19:**
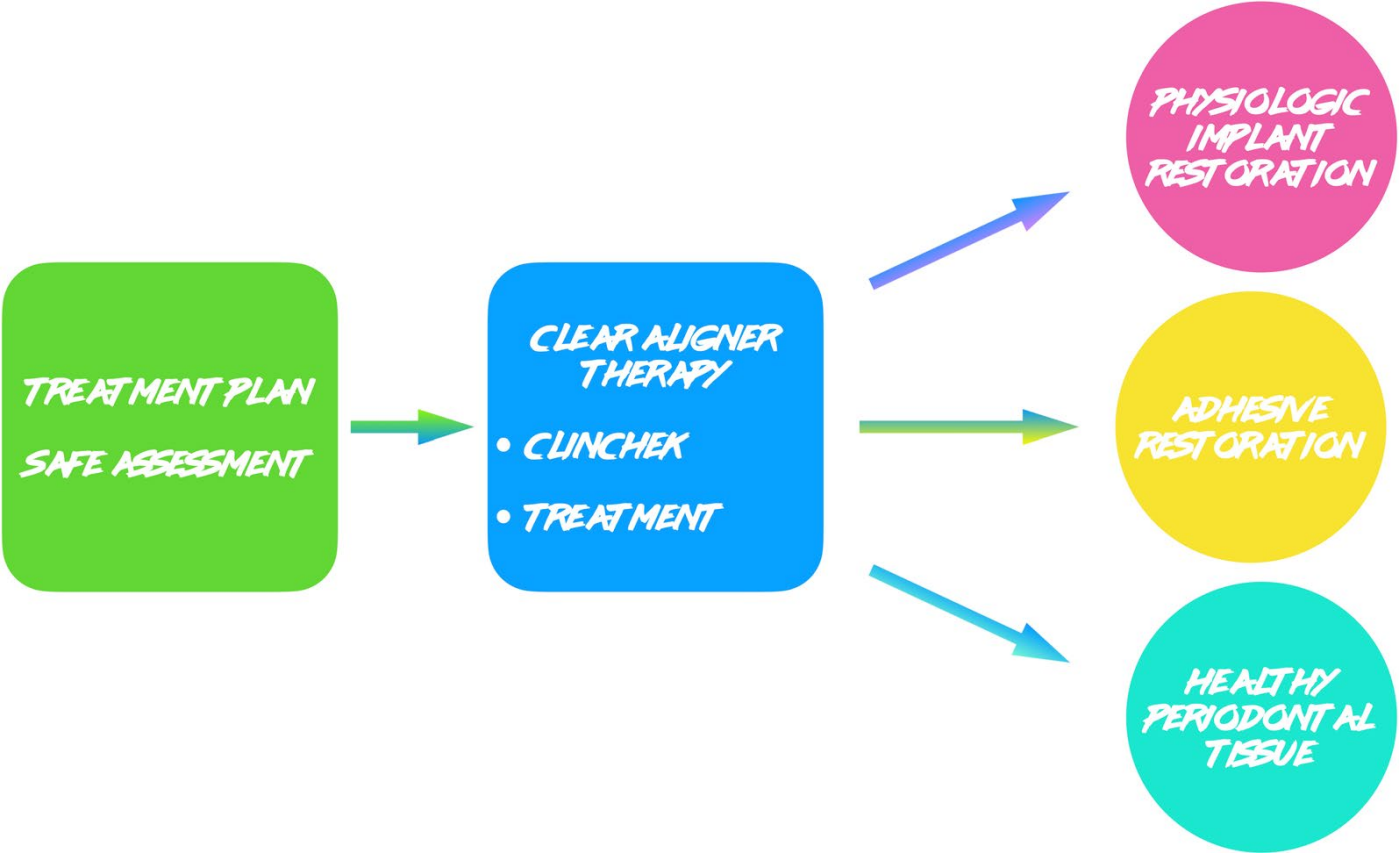
Ideal restorative workflow with CAT

The FGTP is a guide to inter-disciplinary planning, it allows for realistic treatment goals to be set for adults with a vision shared by the whole team. It is also a potential tool to communicate with the patient. It is a comprehensive analysis of the case which can be usefully used to understand the case and elaborate a correct treatment plan especially built for a pre-restorative phase with aligners. The SAFE assessment transposes all these concepts with focus into ortho-restorative cases with CAT. It is a comprehensive assessment which allows general practitioners as well as specialist orthodontists to deeply analyze and plan any cases. CAT is a well-established technique to straighten teeth. It is an alternative to fixed braces able to treat quite every kind of malocclusion [22]. A systematic review stated that CAT is able to align and level the arches in adults [23]. CAT is better experienced especially in adults because of less pain and better quality of life [24]. It could be optimal also for per periodontal tissues: during treatment the opportunity to remove clear aligners makes easy the oral hygiene and determines better periodontal health compared to fixed orthodontic appliances [25]. After treatment, oral hygiene is clearly improved with straight teeth. CAT is extremely complementary in restorative cases: straighten teeth allows and prepares the spaces to perform, when needed, minimally invasive restoration (Fig. 19). Furthermore, in case of implant surgery, it could create the ideal space not only for implant positioning but also for the subsequent restoration. Limitations of aligners in three-dimensional tooth movement are well described in the literature [26], such as extrusion, inclination in vestibular-lingual direction of anterior segment and severe rotation [23, 26–28]. Invisalign Go (Align Technology, San Jose, Calif) is specifically designed for pre-restorative cases, allowing limited movement between second premolar and second premolar in both arches. It is designed especially for restorative dentists to give them the chances to treat a case with inter-disciplinary mindset. To starting using Invisalign Go, every dentist must attend an online or face to face specific training program developed by one of the Authors (R.A.). This program provides a basic level of training for general dentists about clear aligners therapy and offers a continuing mentorship provided by experienced doctors for every step of the treatment, from case selection to retention. Case selection could be one of the major issues for dentist as the system allow to treat a specific range of malocclusion (see Table 3). These criteria could be easily applied to every case by using a specific app (Invisalign Photo Uploader, Align Technology, San Jose, Calif) which immediately evaluate the feasibility of the case with this system through photo analysis. The app guides the clinician to take a specific photo protocol; then, the photos are uploaded and a dedicated software classifies the case as “easy” or “difficult” to treat. If the software evaluates the case as “difficult”, this could be referred to a comprehensive Invisalign provider. Generally, following IOTN classification [29], up to grade 3 cases could be treated however patients who have not completed skeletal growth are not suitable for this kind of treatment. Invisalign Go provides a total of 20 aligners plus two sets of 20 aligners each for refinement. In this paper, the first case required a total of 21 aligners, the second one 24. In the Author’s experience cases treated with Invisalign Go usually present this amount of duration. The Clin Check is a powerful tool to plan and to share with the patient the treatment plan. It helps to build a correct therapeutic alliance between clinicians and patients. The possibility to align teeth is extremely useful in a pre-restorative phase, as elicit the chance to work as much as possible at enamel level, which is a favorable prognostic factor for adhesive restorations [30]. In case 1 the correction of cross bite avoided any restorative intervention on tooth 1.2. In case 2 the Clin Check allowed to measure and create exactly the correct space for tooth 1.2, based on the Bolton analysis (15). An additive restoration was performed to conform the tooth to the contralateral and the correction of the cross bite prevented from occlusal stress and restore function.

**Table 3:** Limits of application of Invisalign Go

Misalignment	Limit
Crowding/spacing	≤ 7 mm
Arch/width expansion	4 mm per arch
Overbite (deep vs. open)	≤ 5 mm total
Midline discrepancy	1 mm
Treatment includes • Number of aligners per arch • Additional aligners	Up to 20 Two sets (1,2)

The preservation of dental tissue has become possible especially with the spreading and the improvement of modern adhesive partial restorations. The merging of these concepts let clinicians to interrupt the cycle of defective restorations [31, 32] that is the cycle of a failed restoration substituted by a larger one, leading to more extensive restorations during years ending in root canal therapy and possibly an implant.

Adhesive dentistry allows to preserve and conserve sound tooth structure: full crown preparation could not be necessary anymore as long as mechanical retention could be substituted by adhesively bonded restoration to the remaining tooth structure [33], increasing the lifespan of the treated tooth. When it comes to an esthetic area, this approach should be definitely stressed. As dental clinicians, we should be aware that our restorations could not last forever [34]: it is mandatory to inform the patient about pros and cons of every treatment plan.

Oral health is a prerequisite of every dental treatment and esthetic should be evaluated as first parameter during planning. The newly developed five-to-five CAT could be extremely useful for general practitioners in multidisciplinary treatment plan in order to straighten teeth, especially in a pre-restorative phase to allow minimally invasive and adhesive restorations. Future research will be useful to validate this approach, evaluating the predictability of the treatment and its impact on periodontal health and survival and success of restorations.

## Data Availability

Not applicable: since the study is a case report, there is not a dataset.

## Abbreviations

CAT: Clear aligner therapy
SAFE: Stability Assessment Function and Ethics
FGTP: Facially Generated Treatment Planning
